# The Worriers' Guild

**DOI:** 10.3201/eid1110.AD1110

**Published:** 2005-10

**Authors:** Philip F. Deaver

**Keywords:** another dimension, poem, worriers’ guild, worry

Today there is a meeting of theWorriers' Guild,and I'll be there.The problems of Earth are

to be discussed

at length

end to end

for five days

end to end

with 1100 countries represented

all with an equal voice

some wearing turbans and smocks

and all the men will speak

and the women

with or without notes

in 38 languages

and nine different species of logic.Outside in the autumn

the squirrels will be

chattering and scampering

directionless throughout the town

becausethey aren't organized yet.

